# Integrative approach for detecting membrane proteins

**DOI:** 10.1186/s12859-020-03891-x

**Published:** 2020-12-21

**Authors:** Munira Alballa, Gregory Butler

**Affiliations:** 1 Department of Computer Science and Software Engineering, Concordia University, Montreal, QC Canada; 2 College of Computer and Information Sciences, King Saud University, Riyadh, Saudi Arabia; 3 Centre for Structural and Functional Genomics, Concordia University, Montreal, QC 24105 Canada

**Keywords:** Membrane, Prediction model, Machine learning, Amino acid composition, Integral membrane proteins, Surface-bound membrane proteins, Transmembrane, Integrative approach

## Abstract

**Background:**

Membrane proteins are key gates that control various vital cellular functions. Membrane proteins are often detected using transmembrane topology prediction tools. While transmembrane topology prediction tools can detect integral membrane proteins, they do not address surface-bound proteins. In this study, we focused on finding the best techniques for distinguishing all types of membrane proteins.

**Results:**

This research first demonstrates the shortcomings of merely using transmembrane topology prediction tools to detect all types of membrane proteins. Then, the performance of various feature extraction techniques in combination with different machine learning algorithms was explored. The experimental results obtained by cross-validation and independent testing suggest that applying an integrative approach that combines the results of transmembrane topology prediction and position-specific scoring matrix (Pse-PSSM) optimized evidence-theoretic *k* nearest neighbor (OET-KNN) predictors yields the best performance.

**Conclusion:**

The integrative approach outperforms the state-of-the-art methods in terms of accuracy and MCC, where the accuracy reached a 92.51% in independent testing, compared to the 89.53% and 79.42% accuracies achieved by the state-of-the-art methods.

## Background

Membrane proteins play essential roles in transport, signaling, adhesion, and metabolism, which positions them as a leading drug target; over half of the current FDA-approved drugs target membrane proteins [1]. Membrane proteins are among the least characterized proteins in terms of their structure and function due to their hydrophobic surfaces and poor conformational stability. Distinguishing membrane proteins can help direct future experiments and provide clues regarding the functions of these proteins.

A major class of membrane proteins are transmembrane proteins. These proteins have one or more transmembrane segments (TMSs) embedded in the lipid bilayer in addition to extramembranous hydrophilic segments that extend into the water-soluble domains on each side of the lipid bilayer. The embedded segments are distinguishable because they contain residues with hydrophobic properties that interact with the hydrophobic (nonpolar) tails of the membrane phospholipids. Other classes of membrane proteins include surface-bound proteins that do not extend into the hydrophobic interior of the lipid bilayer; they are typically bound to the lipid head groups at the membrane surface or attach to other transmembrane proteins. Unlike transmembrane proteins, surface-bound proteins such as peripheral and lipid-anchored proteins do not have TMSs; they are therefore more difficult to distinguish from other globular proteins.

Two distinct approaches, namely, transmembrane topology prediction and membrane structure type prediction, are primarily used to detect membrane proteins. While transmembrane topology tools predict only a subset of membrane proteins (transmembrane proteins), they are applied more often than membrane structure type prediction tools due to the vast number of tools available and because transmembrane proteins constitute a major class of membrane proteins. However, by overlooking other classes of membrane proteins, essential information is lost. By contrast, membrane structure type predictions can be used to detect all classes of membrane proteins. In this work, we focus on detecting membrane proteins of all types and answering this question: *given a protein sequence Q, is it a membrane protein?*

The state-of-the-art tools that have achieved the highest overall performance in predicting all types of membrane proteins are MemType-2L [2] and iMem-2LSAAC [3]. While MemType-2L [2] has been in use for over a decade, it has maintained its popularity due to its simple yet effective methodology. MemType-2L incorporates evolutionary information by representing protein samples with pseudo position-specific scoring matrix (Pse-PSSM) vectors and combining the results obtained from individual optimized evidence-theoretic *k* nearest neighbor (OET-KNN classifiers). By contrast, iMem-2LSAAC uses the split amino acid composition (SAAC) to extract features from protein samples and then support vector machine (SVM) to train the predictor.

MemType-2L is the only accessible tool for the prediction of all types of membrane proteins. When we tested it on a new set of membrane proteins, the accuracy reached only 80%, compared with the estimated accuracy of 92.7% in the original paper. This is because it was trained on the available protein sequences from 2006; and this protein sequence landscape has drastically changed, where a large surge in protein sequence entries has been recorded since then. It is therefore essential to build a new accessible tool that accommodates all membrane data.

The main contributions of this work can be summarized as follows:

- We establish a new benchmark dataset for membrane proteins (*DS-M*).
- We evaluate the performances of traditional transmembrane topology prediction tools on *DS-M* to predict all types of membrane proteins.
- We compare the performances of various machine learning techniques to detect membrane proteins; this comparison involved applying different feature extraction techniques to encode protein sequences and choosing the proper machine learning algorithm to build a model using the extracted vectors.
- We introduce a novel method, *TooT-M*, which integrates different techniques that achieves superior performance compared to all other methods, including the state-of-the-art methods.

### Transmembrane topology prediction

Transmembrane topology prediction methods predict the number of TMSs and their respective positions in the primary protein sequence. Transmembrane proteins are integral membrane proteins (IMPs) that span the lipid bilayer and have exposed portions on both sides of the membrane. It is expected that the portions that span the membrane contain hydrophobic (nonpolar) amino acids, while the portions that are on either side of the membrane consist mostly of hydrophilic (polar) amino acids. The TMSs can have either $\alpha$-helical or $\beta$-barrel structures, so prediction methods are classified into $\alpha$-helix prediction methods and $\beta$-barrel prediction methods.

Previous prediction methods depended solely on simple measurements such as the hydrophobicity of the amino acids [4]. Major improvements were made after the “positive-inside rule” [5] was introduced by Von Heijne, which came from the observation that positively charged amino acids, such as arginine and lysine, tend to appear on the cytoplasmic side of the lipid bilayer. Current methods combine hydrophobicity analysis and the positive-inside rule together with machine learning techniques and evolutionary information.

For example, the membrane protein structure and topology support vector machine MEMSAT–SVM method [6], introduced in 2009, uses four support vector machines (SVMs) to predict transmembrane helices, inside and outside loops, re-entrant helices and signal peptides. In addition, it includes evolutionary information on many homologous protein sequences in the form of a sequence profile. This method outputs predicted topologies ranked by the overall likelihood and incorporates signal peptide and re-entrant helix prediction. The reported accuracy is 89% for the correct topology and location of TM helices and 95% for correct number of TM helices. However, recent studies using experimental data report that MEMSAT–SVM does not perform as well when evaluated on different datasets [7, 8].

State-of-the-art methods use consensus algorithms that combine the outputs from different predictors. The consensus prediction of membrane protein topology (TOPCONS2) method [8] achieved the highest reported prediction accuracy based on benchmark datasets [9]. It successfully distinguishes between globular and transmembrane proteins and between transmembrane regions and signal peptides. In addition, it is highly efficient, making it ideal for proteome-wide analyses. The TOPCONS2 method combines the outputs from different predictors that can also predict signal peptides (namely, Philius [10], PolyPhobius [11], OCTOPUS [12], signal peptide OCTOPUS (SPOCTOPUS) [13], and SCAMPI [14]) into a topology profile where each residue is represented by one of four values: the signal peptide (S), a membrane region (M), the inside membrane (I), or outside membrane (O). Then, a hidden Markov model is used to process the resulting profile and predict the final topology with the highest-scoring state path.

Regarding $\beta$-barrel membrane protein prediction, a variety of methods have been introduced, such as methods that combine statistical propensities [15], k-nearest neighbor (KNN) methods [16], neural networks [17, 18], hidden Markov models [19–22], SVMs [23], and amino acid compositions (AACs) [24, 25]. Approaches based on hidden Markov models have been found to achieve statistically significant performance when compared to other types of machine learning techniques [26]. Major methods for detecting $\beta$-barrel outer membrane proteins are HHomp [27], $\beta$-barrel protein OCTOPUS (BOCTOPUS) [21], and PRED-TMBB2 [22], with reported MCCs of 0.98, 0.93, and 0.92, respectively, when applied to the same dataset. The BOCTOPUS and HHomp techniques are much slower than PRED-TMBB2 [22].

### Prediction of the membrane protein structural type

Methods for predicting membrane type can predict up to eight different membrane protein structural subtypes categorized as single-pass types I, II, III, and IV; multipass transmembrane; glycophosphatidylinositol (GPI)-anchored; lipid-anchored; and peripheral membrane proteins. A comprehensive review by Butts et al. [28] elucidates these methods in detail. Generally, prediction is performed in two stages: the first stage identifies the protein sequence as membrane or nonmembrane, while the second stage differentiates among specific membrane protein subtypes. This research focuses on detecting all membrane proteins, regardless of their type (the first stage).

The MemType-2 [2] predictor was introduced in 2007 by Chou and Shen. It is a two-layer predictor that uses the first layer to identify a query protein as a membrane or nonmembrane protein. Then, if the protein is predicted as a membrane protein, the second layer identifies the structural type from among the eight categories. The MemType-2L predictor incorporates evolutionary information by representing the protein samples with Pse-PSSM vectors and combining the results obtained by OET-KNN classifiers. It achieved an overall accuracy of 92.7% in the membrane detection layer. The reported performance in the first layer is obtained by applying the jackknife test on the provided dataset.

Butts et al. [29] introduced a tool that predicts all types of membrane proteins; it uses statistical moments to extract features from the protein samples and then trains a multilayer neural network with backpropagation to predict the membrane proteins. This tool achieved an overall accuracy of 91.23% when applying the jackknife test on the dataset from Chou and Shen [2], which was a slightly lower performance than the MemType-2L predictor.

The iMem-2LSAAC was introduced in 2017 by Arif et al. [3]. iMem-2LSAAC is a two-layer predictor that uses the first layer to predict whether a query protein is a membrane protein. Then, in the case of membrane proteins, it continues to the second layer to identify the structural category. It utilizes the split amino acid composition (SAAC) to extract the features from the protein samples and then applies an SVM to train the predictor. iMem-2LSAAC achieved an overall accuracy of 94.61% in the first layer when applying the jackknife estimator on their dataset.

## Methods

### Dataset

The latest publicly available benchmark dataset that contains both membrane and nonmembrane proteins was constructed by Chou and Shen [2] and was used to construct the MemType-2L predictor. Their dataset was collected from the Swiss-Prot database version 51.0, released on October 6, 2006. Furthermore, they eliminated proteins with 80% or more similarity in their sequences to reduce homology bias. Chou and Shen’s dataset contains a total of 15,547 proteins, of which 7582 are membrane proteins and 7965 are nonmembrane proteins.

Because of the rapidly increasing sizes of biological databases, we built a new updated dataset, *DS-M*. This dataset was collected from the Swiss-Prot database. The annotated membrane proteins were retrieved by extracting all of the proteins that are located in the membrane, using the following search query: 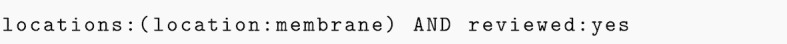


The remainder of the Swiss-Prot entries were designated as nonmembrane proteins.

The sequences in both classes were filtered by adhering to the following criteria:

- *Step 1*: Protein sequences that have evidence “inferred from homology” for the existence of a protein were removed.
- *Step 2*: Protein sequences less than 50 amino acids long were removed, as they could be fragments.
- *Step 3*: Protein sequences that have no Gene Ontology MF annotation or annotation based only on computational evidence (inferred from electronic annotation, IEA) were excluded.
- *Step 4*: Protein sequences with more than 60% pairwise sequence identity were removed via a CD-HIT [30] program to avoid any homology bias.

All sequences from the membrane class and randomly selected sequences from the nonmembrane class were used to form the benchmark dataset. The data were randomly divided (stratified by class) into the training (90%) and testing sets (10%). To further limit homology bias between the training and testing sets, the sequences in the testing set were filtered such that no sequence has more than 30% pairwise identity to any sequence in the training set. The number of sequences in the training and testing datasets are illustrated in Table 1.

The dataset contains samples from different species, with the most sequences coming from *Homo sapiens *(18%), *Arabidopsis thaliana *(14%), *Mus musculus *(11%), *Saccharomyces cerevisiae *(8%), and *Saccharomyces pombe *(6%).

**Fig. 1:**
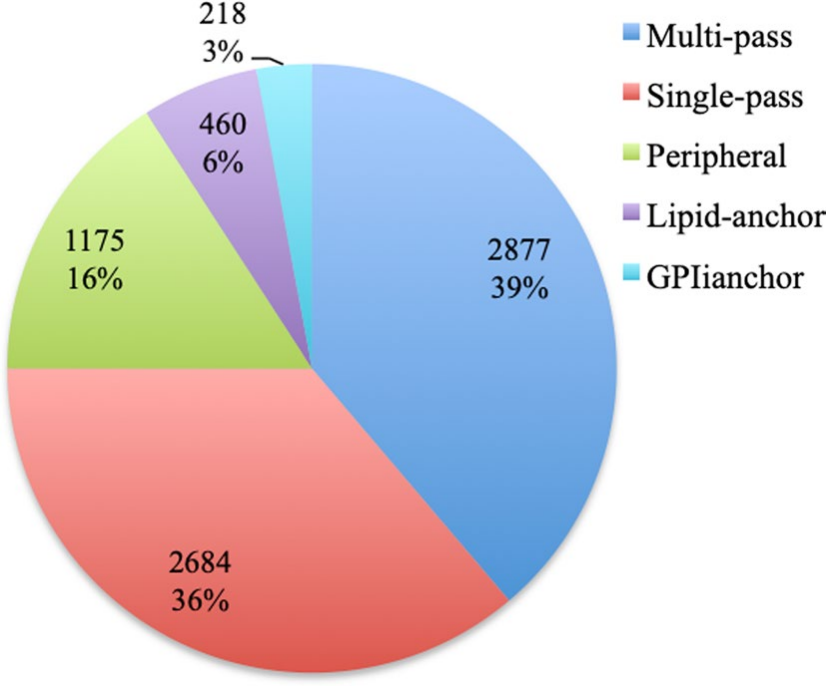
Membrane structural types

Approximately 84% of the membrane data collected have a structural type annotation. Fig. 1 indicates that of the annotated proteins, approximately 75% are transmembrane proteins (single or multipass), while the remainder are peripheral, lipid-anchored, or GPI-anchored proteins.

**Fig. 2:**
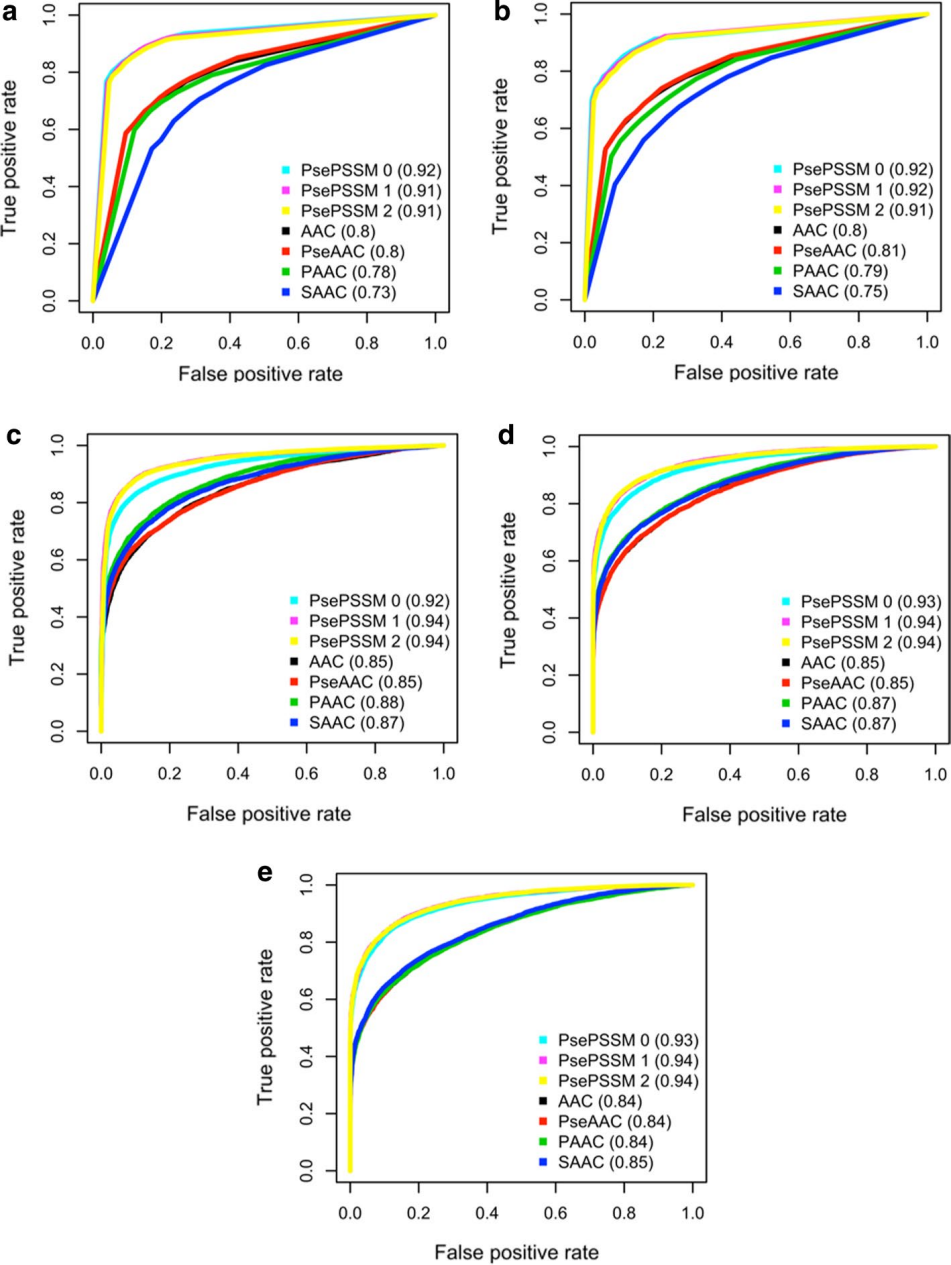
Receiver operating characteristic analysis. Receiver operating characteristic (ROC) curves and the area-under-curve (AUC) scores for each model built using **a** OET-KNN; **b** KNN; **c** SVM; **d** GBM; **e** RF logarithms

**Fig. 3:**
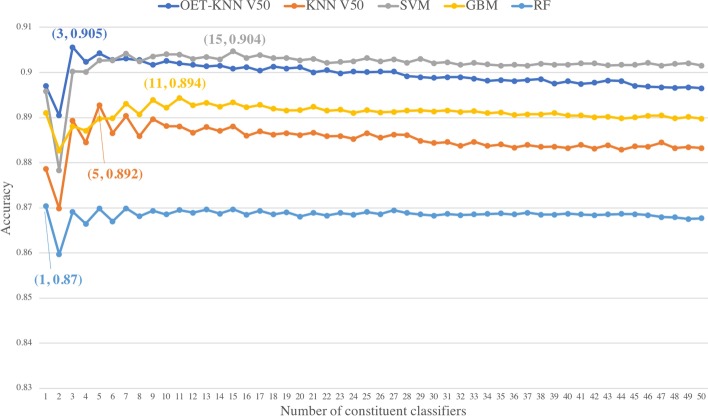
Choice of the optimal constituent classifiers among 50 classifiers. In the pair (*x*, *y*), *x* refers to the number of top-ranked components in the optimal feature set, and *y* refers to the achieved accuracy using those *x* components. The accuracy peaked when the number of top-ranked components were 3, 5, 15, 11, 1 for the OET-KNN V50-, KNN V50-, SVM-, GBM-, and RF-based ensembles, respectively

**Fig. 4:**
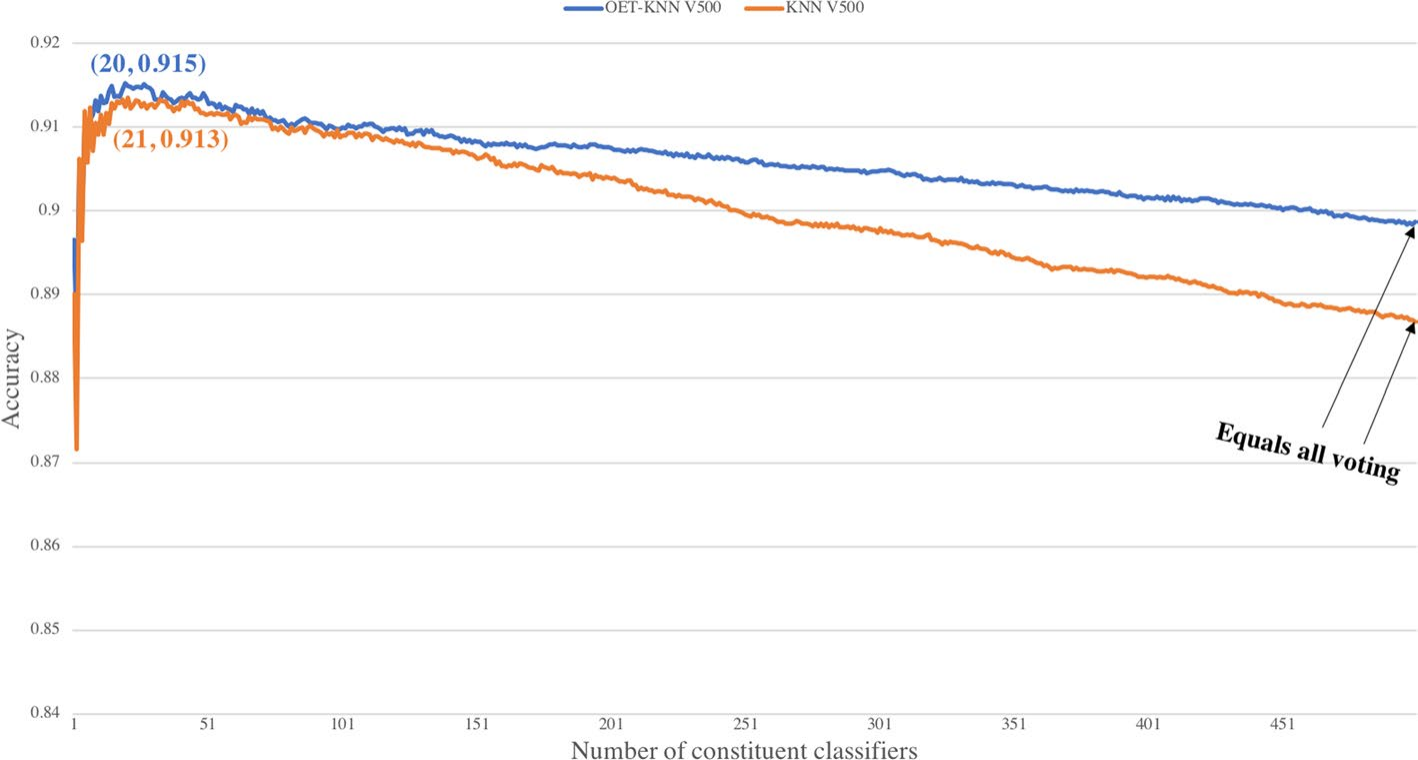
Choice of the optimal constituent classifiers among 500 classifiers. In the pair (*x*, *y*), *x* refers to the number of top-ranked components in the optimal feature set, and *y* refers to the achieved accuracy using those *x* components. The optimal numbers of features for the OET-KNN V500 and KNN V500 ensembles were 20 and 21, respectively. The performance started to deteriorate as more votes were accounted for. Overall, the results suggest that the *selective voting* approach outperforms the *all voting* approach

**Fig. 5:**
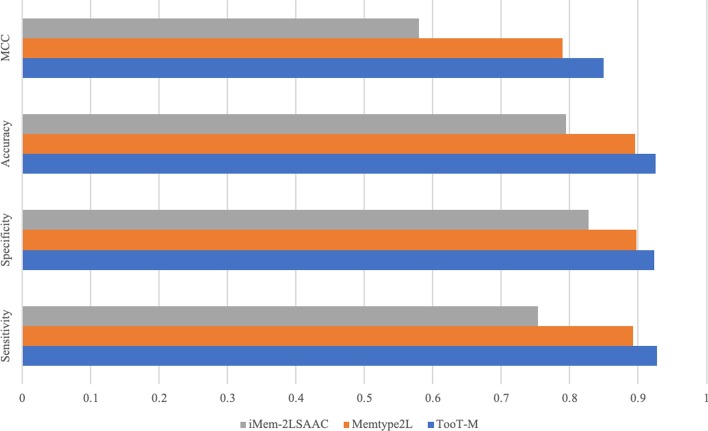
Comparison with other state-of-the-art methods on the *DS-M* dataset

**Fig. 6:**
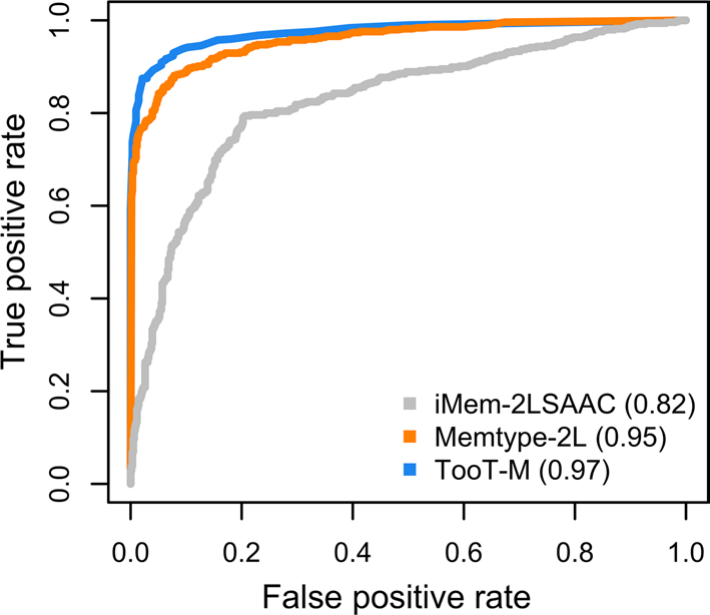
Receiver operating characteristic analysis. ROC curves and the area-under-curve (AUC) scores for TooT-M and the state-of-the-art methods on *DS-M* dataset

**Table 1:** Membrane dataset *DS-M*

Class	Training	Testing	Total
Membrane	7945	495	8440
Nonmembrane	8157	613	8770
Total	16,102	1108	17,210

### Topology prediction tools

A protein is regarded as a membrane protein if at least one TMS is detected. With respect to $\alpha$-helical transmembrane proteins, three tools were applied. First, TOPCONS2 [8] which is considered to be the state-of-the-art method and known for its ability to distinguish signal peptides from transmembrane regions, TOPCONS2 results were obtained through its available web server. The second tool is HMMTOP [31], which is a highly efficient tool commonly used in the literature, HMMTOP results were also obtained through its web server. The third tool is TMHMM [32], also commonly applied in the literature, and its results were obtained from its web server.

**Table 2:** LOOCV performance of the individual models

Encoding	ML algorithm	Sensitivity	Specificity	Accuracy	MCC
AAC	OET-KNN	71.34	81.08	76.28	0.5271
	KNN	75.72	74.87	75.29	0.5058
	SVM	70.96	83.47	77.30	0.5492
	GBM	71.86	83.75	77.89	0.5606
	RF	68.11	85.13	76.73	0.5409
PseAAC	OET-KNN	73.05	81.38	77.27	0.5465
	KNN	74.24	79.38	76.84	0.5370
	SVM	70.59	83.98	77.37	0.5511
	GBM	74.99	86.07	80.60	0.6149
	RF	68.84	84.86	76.95	0.5446
PAAC	OET-KNN	68.94	72.09	70.53	0.4105
	KNN	72.96	66.26	69.57	0.3930
	SVM	76.15	84.22	80.24	0.6060
	GBM	71.33	85.01	77.84	0.5661
	RF	71.00	81.67	76.41	0.5301
SAAC	OET-KNN	66.63	72.88	69.80	0.3960
	KNN	69.75	68.81	69.28	0.3856
	*SVM*	*72.51*	*85.85*	*79.27*	*0.5895*
	GBM	73.90	85.95	80.00	0.6034
	RF	67.82	87.02	77.54	0.5595
Pse-PSSM, $\lambda =0$	OET-KNN	86.57	92.75	89.70	0.7953
	KNN	85.22	90.44	87.86	0.7580
	SVM	83.23	90.05	86.68	0.7350
	GBM	83.41	90.45	86.98	0.7409
	RF	79.45	92.53	86.08	0.7269
Pse-PSSM, $\lambda =1$	OET-KNN	85.92	91.79	88.89	0.7788
	KNN	85.89	89.06	87.50	0.7501
	SVM	86.75	92.22	89.52	0.7912
	GBM	85.00	92.19	88.64	0.7744
	RF	79.86	93.66	86.85	0.7433
Pse-PSSM, $\lambda =2$	OET-KNN	85.51	91.90	88.75	0.7762
	KNN	85.65	88.28	86.98	0.7397
	SVM	86.83	92.06	89.48	0.7904
	GBM	84.86	91.72	88.34	0.7682
	RF	79.80	93.70	86.84	0.7432

This table shows microaverage LOOCV performance of the different protein encodings on different machine learning algorithms. The SAAC with SVM, highlighted in italics, reflects the LOOCV performance of the iMem-2LSAAC method [3] on *DS-M*. Only the Pse-PSSMs where $\lambda \in (0, 1, 2)$ are shown here; the complete performance of all the Pse-PSSMs ($\lambda \in (0, \ldots , 49) )$ can be found in Additional file 1

**Table 3:** Performances of the *all voting* ensemble classifiers on the main dataset

Algorithm	Sensitivity	Specificity	Accuracy	MCC
*OET-KNN V500*	*85.10*	*94.51*	*89.86*	*0.8004*
OET-KNN V50	85.61	93.57	89.64	0.7950
KNN V500	85.50	91.77	88.68	0.7747
KNN V50	86.19	90.40	88.32	0.7669
SVM	86.48	93.72	90.15	0.8047
GBM	84.52	93.32	88.98	0.7820
RF	79.38	93.95	86.76	0.7423

*all voting* with OET-KNN V500, highlighted in italics, reflects the LOOCV performance of the MemType-2L method on *DS-M*

**Table 4:** Performances of the *selective voting* ensemble classifiers on the main dataset

Algorithm	Sensitivity	Specificity	Accuracy	MCC
*OET-KNN V500*	*88.99*	*94.00*	*91.53*	*0.8314*
OET-KNN V50	86.58	94.43	90.56	0.8133
KNN V500	89.01	93.63	91.35	0.8280
KNN V50	86.55	91.92	89.27	0.7863
SVM	87.12	93.72	90.46	0.8107
GBM	85.30	93.45	89.44	0.7909
RF	80.19	93.71	87.04	0.7468

*Selective voting* with OET-KNN V500, highlighted in italics, refers to the method that achieved the highest MCC and is the method utilized in *TooT-M*

Regarding $\beta$-barrel transmembrane proteins, we applied PRED-TMBB2 [22], which shows comparable performance to the state-of-the-art $\beta$-barrel predictors but is much more efficient in terms of the runtime [22], The results of PRED-TMBB2 were obtained from its available web server.

### Protein sequence encoding

After establishing the dataset, it is necessary to find the best representation of the protein sequences used to train the prediction engine. Generally, there are two options: sequential or discrete representations [2]. In sequential representations, a sample protein is represented by its amino acid sequence and then used in a similarity search-based tool such as BLAST [33]. A major drawback of relying on the similarity is that it fails when proteins with the same function share a low sequence similarity. In discrete representations, a sample protein is represented by a set of discrete numbers that are usually the result of feature engineering. In this study, we encoded the protein sequences using the AAC, PAAC, and PseAAC baseline compositions. In addition, we applied the Pse-PSSM and SAAC as described below.

**Table 5:** Transmembrane topology prediction performance on the training dataset

Tool	Sensitivity	Specificity	Accuracy	MCC
HMMTOP	72.71	84.60	78.73	0.5777
*TOPCONS2*	*69.86*	*99.77*	*85.01*	*0.7318*
TMHMM	68.61	97.14	83.06	0.6878
PRED-TMBB2	41.73	55.48	48.70	− 0.0281

TOPCONS2, highlighted in italics, is the tool that achieved the highest MCC and is the method utilized in *TooT-M*

**Table 6:** *TooT-M* LOOCV performance

Method	Sensitivity	Specificity	Accuracy	MCC
Selective voting OET-KNN V500	89.01	93.63	91.35	0.8280
TOPCONS2	69.86	99.77	85.01	0.7318
*TooT-M*	*91.47*	*94.90*	*93.21*	*0.8645*

This table shows the LOOCV performance of *TooT-M*, which integrates the predictions from the constituent classifiers of the *selective voting* OET-KNN V500 ensemble and TOPCONS2 through weighted voting

**Table 7:** Comparison with other state-of-the-art methods on the *DS-M* dataset

Method	Sensitivity	Specificity	Accuracy	MCC
*TooT-M*	*92.73*	*92.33*	*92.51*	*0.85*
MemType-2L [2]	89.29	89.72	89.53	0.79
iMem-2LSAAC [3]	75.35	82.71	79.42	0.58

This table compares the performance of *TooT-M* integrative approach with other state-of-the-art methods on the *DS-M* dataset. The highest performance in each metric is highlighted in italics. *TooT-M* outperformed the state-of-the-art methods across all metrics

**Table 8:** Comparison with the iMem-2LSAAC predictor on the DS1 dataset

Method	Sensitivity	Specificity	Accuracy	MCC
*TooT-M*	98.09	*96.80*	*97.43*	*0.94*
iMem-2LSAAC	*98.23*	91.17	94.61	0.89

This table compares the performance of *TooT-M* with the state-of-the-art iMem-2LSAAC predictor [3] on the same dataset, DS1. The best performance for each metric is highlighted in italics. *TooT-M* achieved a higher specificity, accuracy and MCC than iMem-2LSAAC

**Table 9:** Comparison with the MemType-2L predictor on the DS2 dataset

Method	Sensitivity	Specificity	Accuracy	MCC
*TooT-M*	*92.71*	*94.4*	*93.57*	*0.87*
MemType-2L	91.00	*94.4*	92.7	0.85

This table compares the performance of *TooT-M* with the state-of-the-art MemType-2L predictor [2] on the same dataset, DS2. The best performance for each metric is highlighted in italics. *TooT-M* achieved a higher sensitivity, accuracy and MCC than MemType-2L and the same specificity

#### Amino acid composition (AAC)

The AAC is the normalized occurrence frequency of each amino acid. The fractions of all 20 natural amino acids are calculated by:

1 $$\begin{aligned} c_i = \frac{F_i}{L} \qquad i=(1,2,3,\ldots ,20) \end{aligned}$$

where $F_i$ is the frequency of the $i{\mathrm{th}}$ amino acid and *L* is the length of the sequence. Each protein’s AAC is represented as a vector of size 20 as follows:

2 $$\begin{aligned} AAC(P) = \left[ c_{1} , c_{2} , c_{3} , \ldots , c_{20} \right] \end{aligned}$$

where $c_{i}$ is the composition of the $i{\mathrm{th}}$ amino acid.

#### Pair amino acid composition (PAAC)

The PAAC has an advantage over the AAC because it encapsulates information about the fraction of the amino acids as well as their order. It is used to quantify the preference of amino acid residue pairs in a sequence. The PAAC is calculated by:

3 $$\begin{aligned} d_{i,j} = \frac{F_{i,j}}{L-1} \qquad i,j=(1,2,3,\ldots ,20) \end{aligned}$$

where $F_{i,j}$ is the frequency of the $i{\mathrm{th}}$ and $j{\mathrm{th}}$ amino acids of a pair (dipeptide) and *L* is the length of the sequence. Similar to the AAC, the PAAC is represented as a vector of size 400 as follows:

4 $$\begin{aligned} PAAC(P) = \left[ d_{1,1} , d_{1,2}, d_{1,3} , \ldots , d_{20,20} \right] \end{aligned}$$

where $d_{i,j}$ is the dipeptide composition of the $i{\mathrm{th}}$ and $j{\mathrm{th}}$ amino acids.

#### Pseudo-amino acid composition (PseAAC)

The PseAAC was proposed in 2001 by Chou [34] and showed a remarkable improvement in the prediction quality when compared to the conventional AAC. PseAAC is a combination of the 20 components of the conventional AAC and a set of sequence-order correlation factors that incorporate some biochemical properties. Given a protein sequence of length *L*,

5 $$\begin{aligned} R_1 R_2 R_3 R_4 \ldots R_L \end{aligned}$$

a set of descriptors called sequence-order-correlated factors are defined as follows:

6 $$\begin{aligned} \left\{ \begin{array}{c} \theta _1 = \displaystyle \frac{1}{L-1} \sum _{i=1}^{L-1} \Theta (R_i,R_{i+1}) \\ \theta _2 = \displaystyle \frac{1}{L-2} \sum _{i=1}^{L-2} \Theta (R_i,R_{i+2}) \\ \theta _3 = \displaystyle \frac{1}{L-3} \sum _{i=1}^{L-3} \Theta (R_i,R_{i+3}) \\ . \\ . \\ . \\ \theta _\lambda = \displaystyle \frac{1}{L-\lambda } \sum _{i=1}^{L-\lambda } \Theta (R_i,R_{i+\lambda }) \\ \end{array} \right. \end{aligned}$$

The parameter $\lambda$ is chosen such that $(\lambda <L)$. The correlation function is given by:

7 $$\begin{aligned} {\begin{matrix} \Theta (R_i,R_j)=&{} \displaystyle \frac{1}{3} \left\{ [ H_1(R_j) - H_1(R_i) ]^2 +[ H_2(R_j) - H_2(R_i) ]^2 \right. \\ &{}\left. + [ M(R_j) - M(R_i) ]^2 \right\} \end{matrix}} \end{aligned}$$

where $H_1 (R_i)$ is the hydrophobicity value, $H_2 (R_i)$ is the hydrophilicity value, and $M(R_i)$ is the side-chain mass of the amino acid $R_i$. These quantities were converted from the original hydrophobicity value, the original hydrophilicity value and the original side-chain mass by a standard conversion formula as follows:

8 $$\begin{aligned} H_1 (R_i) = \frac{H^\circ _1 (R_i) - \displaystyle \frac{1}{20} \sum _{k=1}^{20} H^\circ _1 (R_k) }{\sqrt{\displaystyle \frac{\displaystyle \sum _{y=1}^{20} \left[ H^\circ _1 (R_y) - \frac{1}{20} \sum _{k=1}^{20} H^\circ _1 (R_k) \right] ^2}{20} } } \end{aligned}$$

where $H^\circ _1 (R_i)$ is the original hydrophobicity value for amino acid $R_i$ and can be taken from the work of Tanford [35]; $H^\circ _2 (R_i)$ and $M^\circ (R_i)$ are converted to $H_2 (R_i)$ and $M(R_i)$, respectively, in the same way. The original hydrophilicity value $H^\circ _2 (R_i)$ for amino acid $R_i$ can be obtained from Hopp and Woods [36]. The mass $M^\circ (R_i)$ of the $R_i$ amino acid side chain can be obtained from any biochemistry textbook. PseAAC is represented as a vector of size $(20+ \lambda )$ as follows:

9 $$\begin{aligned} PseAAC(P) = \left[ \displaystyle s_{1} ,\ldots , s_{20} , s_{21} , \ldots ,s_{20+ \lambda } \right] \end{aligned}$$

where $s_{i}$ is the pseudo-AAC as follows:

10 $$\begin{aligned} s_{i} = \left\{ \begin{array}{cc} \displaystyle \frac{f_i}{\sum _{r=1}^{20} f_r+ \omega \sum _{j=1}^{\lambda } \theta _j} &{} 1 \le i \le 20 \\ \displaystyle \frac{\omega \theta _{i-20}}{\sum _{r=1}^{20} f_r+ \omega \sum _{j=1}^{\lambda } \theta _j} &{} 20 < i \le 20 + \lambda \end{array} \right. \end{aligned}$$

where ${f_i}$ is the normalized occurrence frequency of the *ith* amino acid in the protein sequence, $\theta _j$ is the $j{\mathrm{th}}$ sequence-order-correlated factor calculated from Equation 6, and $\omega$ is a weight factor for the sequence-order effect. The weight factor $\omega$ puts weight on the additional PseAAC components with respect to the conventional AAC components. The user can select any value from 0.05 to 0.7 for the weight factor. The default value of 0.05 given by Chou [34] was applied in this study.

#### Pseudo position-specific scoring matrix (Pse-PSSM)

We adopted the Chou and Shen [2] protein-encoding strategy, Pse-PSSM. The Pse-PSSM is built by first performing a Position-Specific Iterative BLAST (PSI-BLAST) [33] search on a protein sequence **P** using the Swiss-Prot database (3 iterations, e-value cutoff of 0.001) and retrieving the PSSM profile:

11 $$\begin{aligned} P_{PSSM}= \begin{bmatrix} E_{1\rightarrow 1} &{}\quad E_{1\rightarrow 2} &{}\quad E_{1\rightarrow 3} &{}\quad \dots &{}\quad E_{1\rightarrow 20} \\ \vdots &{}\quad \vdots &{}\quad \vdots &{}\quad &{}\quad \vdots \\ E_{i\rightarrow 1} &{}\quad E_{i\rightarrow 2} &{}\quad E_{i\rightarrow 3} &{}\quad \dots &{}\quad E_{i\rightarrow 20} \\ \vdots &{}\quad \vdots &{}\quad \vdots &{}\quad &{}\quad \vdots \\ E_{L\rightarrow 1} &{}\quad E_{L\rightarrow 2} &{}\quad E_{L\rightarrow 3} &{}\quad \dots &{}\quad E_{L\rightarrow 20} \end{bmatrix} \end{aligned}$$

$P_{PSSM}$ has *L* rows (a row for each position in protein sequence **P**) and 20 columns (one for each amino acid). Each element $E_{i\rightarrow j}$ represents the score for the substitution of the amino acid in the $i{\mathrm{th}}$ position of the protein sequence to the amino acid of type *j* in the evolution process. Since the number of columns in the PSSM depends on the length of the protein sequence **P**, the Pse-PSSM first standardizes the PSSM scores so that they have a mean value of zero over the 20 amino acids and then uses the following uniform size vector to represent protein sequence **P**:

12 $$\begin{aligned} P_{Pse-PSSM}^{\lambda }= [ \overline{E}_1 , \overline{E}_2, \ldots ,\overline{E}_{20}, G_1^{\lambda }, G_2^{\lambda } , \ldots , G_{20}^{\lambda }] \end{aligned}$$

where $\overline{E}_j$ and $G_j^{\lambda }$ are defined as follows:

13 $$\begin{aligned} \overline{E}_j= & {} \frac{1}{L} \sum ^{L}_{i=1} E_{i\rightarrow j} \qquad (j= 1,2, \ldots 20) \end{aligned}$$

14 $$\begin{aligned} G_j^{\lambda }= & {} \frac{1}{L-\lambda } \sum ^{L-\lambda }_{i=1} [E_{i\rightarrow j}- E_{(i + \lambda ) \rightarrow j} ]^2 \qquad (j= 1,2, \ldots 20) \end{aligned}$$

$\lambda$ is chosen such that $(\lambda <L)$. Since the shortest protein in our dataset is 50 amino acids long, we considered all $\lambda \in (0, \ldots , 49)$, and the performance of each encoding was evaluated separately.

#### Split amino acid composition (SAAC)

The concept of SAAC was first reported by Hayat et al. [37]. The motivation behind this concept is that sometimes the most important information is concealed in fragments, and when calculating the AAC for the whole sequence, such information may be masked by noisy, irrelevant information. The SAAC is the sequence encoding used by iMem-2LSAAC, a state-of-the-art predictor of membrane proteins [3].

In SAAC, a protein sequence is divided into segments, and the AAC is computed for each segment separately. Here, we followed the same partitioning described for iMem-2LSAAC [3]: the sequence is divided into three sections, namely, the first 25 amino acids of the N terminus, the last 25 amino acids of the C terminus, and the region between these sections. Each protein is then represented by a vector of size 60, as follows:

15 $$\begin{aligned} SAAC(P) = \left[ c_{1}^{N} , c_{2}^{N} , \ldots c_{20} ^{N}, c_{1} , c_{2} , \ldots c_{20}, c_{1}^{C} , c_{2}^{C} , \ldots c_{20} ^{C} \right] \end{aligned}$$

where $c_i^{N}$, $c_i$, and $c_i^{C}$ are the normalized occurrence frequencies of the $i{\mathrm{th}}$ amino acid in the N terminus, between the two termini, and C terminus segments, respectively.

### Machine learning algorithms

#### K-nearest neighbor (KNN)

KNN is a simple and effective classification algorithm. It is a type of instance-based learning, where all computations are deferred until prediction time. The KNN algorithm assigns a class to an unclassified object X based on the class represented by the majority of its KNNs in the training set vectors. If K = 1, the class of object X will be the class of its nearest neighbor. The choice of K is key to the quality of the KNN prediction engine; we found that the performance started to deteriorate when $K > 10$. We also found that fusing the results of 10 individual classifiers, where $K \in (1, \ldots , 10)$ through majority voting, achieved the highest accuracy and was adopted for the KNN models. We applied the KNN algorithm as implemented by the *class* library in R (version 7.3-15).

#### Optimized evidence-theoretic k-nearest neighbor (OET-KNN)

OET-KNN algorithm is a modification of the traditional KNN algorithm and has been shown to be highly powerful in statistical prediction [38]. It has been used by one of the most powerful membrane predictors, MemType-2L. The OET-KNN algorithm is based on the Dempster-Shafer theory of belief functions [38], wherein each neighbor in a pattern to be classified is regarded as evidence supporting certain hypotheses concerning the class membership of that object. As with the KNN algorithm, any constructed OET-KNN model is an ensemble of multiple OET-KNN classifiers, each with different values of $K \in (1, \ldots , 10)$. The final class was determined through majority voting. We used the OET-KNN algorithm as implemented in R by the *evclass* library (version 1.1.1).

#### Support vector machine (SVM)

SVMs are a powerful machine learning tool used in many biological prediction tools. SVMs aim at solving classification problems by finding appropriate decision boundaries between different classes. In relation to nonlinearly separable data, the kernel trick can be used to transform nonlinear data into a higher-dimensional space where optimal boundaries can be found in an efficient, less computationally expensive process compared to the explicit computations of the coordinates. We used an SVM with a radial basis function (RBF) kernel as implemented by the R *e1071* library (version 1.6-8). The best combination of the C and $\gamma$ parameters was determined by utilizing a grid search approach.

#### Gradient-boosting machine (GBM)

GBMs are a machine learning technique that produces a strong model by assembling weak prediction models, usually decision trees. They use gradient boosting by iteratively training new models based on the weak points of the previous models. While not commonly applied in biological predictions, GBMs have been demonstrated to be one of the most powerful techniques on the popular machine learning competition website Kaggle (kaggle.com). Here, we used the *xgboost* library (version 0.81.0.1), which is a fast and efficient implementation of the gradient-boosting framework in R.

#### Random forest (RF)

RF is an ensemble method for supervised learning that operates by composing multiple uncorrelated decision trees. The goal is to improve accuracy and avoid over-fitting by relying on a collection of decision models instead of a single one.

We applied the RF algorithm as implemented by *caret* [39] package (version 6.0-86) in R. The number of variables randomly sampled as candidates at each split (mtry) was determined through a grid search approach.

### Ensemble classifier

#### All voting

Let $C_{i}^{ML}$ be a classifier built using the machine learning algorithm ML $\in$ {KNN, OET-KNN, SVM, GBM, RF}, in which the protein samples are represented by Pse-PSSM, with $\lambda =i$ and $i \in (0, \ldots , 49)$; each classifier is constructed as described in Machine learning algorithms section.

In addition, let $C_{i,k}^{ML}$ be a classifier built using the machine learning algorithm ML $\in$ {KNN, OET-KNN} in which the protein samples are represented by Pse-PSSM, with $\lambda =i$ and $i \in (0, \ldots , 49)$; and the parameter K that refers to number of neighbors equals *k* and $k \in (1, \ldots , 10)$.

In *all voting*, we evaluated the following seven different ensembles:

- *SVM-based ensemble:* obtains the results from 50 SVM-based classifiers ($C_{0}^{SVM},C_{1}^{SVM} \dots C_{49}^{SVM}$) and combines them through a voting mechanism, where the class that receives the most votes is chosen by the ensemble classifier.
- *GBM-based ensemble:* obtains the results from 50 GBM-based classifiers ($C_{0}^{GBM},C_{1}^{GBM} \dots C_{49}^{GBM}$) and combines them through the same voting mechanism as above.
- *RF-based ensemble:* obtains the results from 50 RF-based classifiers ($C_{0}^{RF},C_{1}^{RF} \dots C_{49}^{RF}$) and combines them through the same voting mechanism as above.
- *KNN V50-based ensemble:* obtains the results from 50 KNN-based ensemble classifiers ($C_{0}^{KNN},C_{1}^{KNN} \dots C_{49}^{KNN}$) and combines them through the same voting mechanism.
- *KNN V500-based ensemble:* obtains the results from 500 KNN-based classifiers (50 for different values of $\lambda$ multiplied by 10 for different values of *K*; $C_{0,1}^{KNN},C_{0,2}^{KNN} \dots C_{49,10}^{KNN}$) and combines them through the same voting mechanism.
- *OET-KNN V50-based ensemble:* obtains the results from 50 OET-KNN-based ensemble classifiers ($C_{0}^{OET-KNN},C_{1}^{OET-KNN} \dots C_{49}^{OET-KNN}$) and combines them through the same voting mechanism.
- *OET-KNN V500-based ensemble:* obtains the results from 500 OET-KNN-based classifiers (50 for different values of $\lambda$ multiplied by 10 for different values of *K*; $C_{0,1}^{OET-KNN},C_{0,2}^{OET-KNN} \dots$ $C_{49,10}^{OET-KNN}$) and combines them through the same voting mechanism; this is the MemType-2L approach [2].

#### Selective voting

For each ensemble in *all voting*, rather than fusing the predictions from all of the individual predictors, here, the optimal subset of predictions (i.e., the output of the constituent classifiers) is selected so that they have minimal redundancy and maximal relevance with the target class. To accomplish this task, we first ranked the features using the minimum redundancy maximum relevance (mRMR) algorithm [40], as implemented by the R *mRMRe* library (version 2.1.0), and then utilized incremental feature selection [41] to choose the optimal subset.

To quantify both the relevance and redundancy, *mRMRe* uses a linear approximation based on correlation such that mutual information (MI) between two variables $c_i$, $c_j$ is estimated as:

16 $$\begin{aligned} MI(c_i,c_j)= - \frac{1}{2} ln(1- \rho (c_i,c_j)^2) \end{aligned}$$

$\rho$ is the correlation coefficient between $c_i$ and $c_j$.

Let *y* be the target class and $X= (c_1,c_2,\dots ,c_n)$ be the set of *n* input features, i.e., the set of constituent classifiers output in *all voting*. The mRMR method ranks the features in *X* by maximizing the MI with *y* (maximum relevance) and minimizing the average MI with all the previously selected variables (minimum redundancy). A list of selected features, denoted by *S*, is initialized with $c_i$, the feature with highest MI with the target variable such that:

17 $$\begin{aligned} c_i= \mathop {{{\,\mathrm{arg\,max}\,}}}\limits _{c_i \in X} MI(c_i, y) \end{aligned}$$

Next, another feature, $c_j$, is added to *S* by choosing the feature that has the highest relevance with the output variable and the lowest redundancy with the previously selected features, utilizing the mutual information difference (MID) scheme:

18 $$\begin{aligned} c_j= \max _{c_j \in \Omega S} \left[ MI(c_j,y) - \frac{1}{|S|} \sum _{c_i \in S} MI(c_j,c_i)\right] \end{aligned}$$

$\Omega S$ denotes the set of features that are not yet added to *S*. This is continued until all of the features in X are added to *S*:

19 $$\begin{aligned} S=(c^\prime _1, c^\prime _2,\dots ,c^\prime _n) \end{aligned}$$

$c^\prime _i$ denotes the feature with the $i{\mathrm{th}}$ rank. Next, we utilized incremental feature selection [41] to choose the optimal subset. Incremental feature selection constructs *n* sets by adding one component at a time in an ascending order, with the $i{\mathrm{th}}$ given as:

20 $$\begin{aligned} s_i= \{c^\prime _1,c^\prime _2 \dots c^\prime _i\} \qquad (1 \le i \le n) \end{aligned}$$

The set with the highest accuracy is then selected for *selective voting*.

### Performance measurement

The performances of the different prediction models were evaluated using jackknife test, also known as leave-one-out cross-validation (LOOCV), in which each sample in the training dataset is predicted based on the rules derived from all of the other samples except the one being predicted; this procedure is repeated so that each sample is used once for validation.

The LOOCV approach was applied to evaluate the state-of-the-art methods of the all-type membrane predictors iMem-2LSAAC [3] and MemType-2L [2], and since the performance of the LOOCV approach does not vary with different runs, it was chosen here.

Furthermore, we evaluated the performance of the model that achieved the highest performance during LOOCV using an independent testing set and compared it to those achieved by the models built with the state-of-the-art methods. Four main evaluation metrics were considered: the sensitivity, specificity, accuracy, and MCC. The sensitivity indicates the proportion of positive samples that are correctly identified.

21 $$\begin{aligned} Sensitivity = \frac{TP}{TP+FN} \end{aligned}$$

The specificity measures the proportion of negative samples that are correctly identified.

22 $$\begin{aligned} Specificity = \frac{TN}{TN+FP} \end{aligned}$$

The accuracy is the number of correct predictions divided by the total number of predictions.

23 $$\begin{aligned} Accuracy = \frac{TP+TN}{TP+FN+TN+FP} \end{aligned}$$

The MCC measures the quality of a binary classifier and returns a value in the range from 1 to ${-}$ 1, where 1 indicates a perfect prediction, 0 represents prediction no better than random, and ${-}$ 1 implies total disagreement between the prediction and observation.

24 $$\begin{aligned} MCC = \frac{(TP\times TN - FP \times FN)}{\sqrt{(TP + FP) \times (TP + FN) \times (TN + FP) \times (TN + FN) }} \end{aligned}$$

In addition, the receiver operating characteristic (ROC) curve was used to evaluate the generalization performance of different models. The area-under-curve (AUC) value was used as a quantitative measure of the robustness of the model. AUC ranges in value from 0 to 1, where 0 indicates a complete disagreement between the prediction and observation, 0.5 represents no better than random prediction, and 1 indicates a perfect prediction.

## Experimental design

The first experiment encodes protein sequences using different methods and uses the generated vectors as input to train different models based on the KNN, OET-KNN, SVM, GBM and RF algorithms; the performances of different models are evaluated on the training set using LOOCV. The second experiment evaluates the two ensemble approaches, *all voting* and *selective voting*, and compares their performances. The third experiment evaluates the performances of the HMMTOP [31], TMHMM [32], TOPCONS2 [8] and PRED-TMBB2 [22] topology prediction tools for detecting all membrane types. Finally, the last experiment integrates the prediction achieved by the best-performing topology prediction tool with the best-performing ensemble in the second experiment; we refer to this integrative approach as *TooT-M*.

In all the abovementioned experiments, only the training set is used to choose the best model/tool. The best-performing method in all of the experiments is chosen as our membrane predictor, and ultimately, its performance is tested on the independent testing set and compared to that achieved by the state-of-the-art methods.

## Results and discussion

### Evaluation of different protein encodings

The LOOCV performances of the baseline encodings AAC, PAAC, and PseAAC, in addition to SAAC, which is utilized by iMem-2LSAAC [3], and the Pse-PSSM utilized by MemType-2L [2] on different machine learning algorithms are illustrated in Table 2. Only the Pse-PSSMs where $\lambda \in (0,1,2)$ are presented here; the rest have comparable performances and are found in Additional file 1. The fivefold and tenfold cross-validation showed consistent results with that of LOOCV, and are presented in Additional file 1.

Since the data are balanced, we focused on the accuracy when comparing the performance of the different models. The encoding extraction techniques can be divided into two primary groups: techniques that extract features solely from a protein sequence, such as AAC, PAAC, PseAAC, and SAAC, and the Pse-PSSM technique that incorporates evolutionary information. Among those techniques that extract features from the protein sequence alone, PseAAC in combination with GBM achieved the highest performance, with an overall validation accuracy of 80.60%, followed by PAAC and SVM, for which the overall accuracy reached 80.28%. The SAAC encoding method used by iMem-2LSAAC [3] was not superior to the other feature extractors, and it reached its highest overall accuracy (80.00%) with the GBM model.

The encoding technique that integrates evolutionary information in the form of Pse-PSSMs for all $\lambda \in (0, \ldots , 49)$ consistently achieved higher accuracy by an average of 11% relative to the methods that rely solely on the protein sequences of individual samples. The highest accuracy reached 89.70%, and was achieved by OET-KNN where the protein samples were encoded using Pse-PSSM $\lambda =0$. On the other hand, when the protein samples were encoded using Pse-PSSM $\lambda \in (1, \ldots , 49)$, the SVM-based models outperformed the models based on the OET-KNN, KNN, GBM and RF algorithms.

To further assess the performance of different encodings, Fig. 2 shows the ROC curve and the value of AUC of each model, and indicates that models with Pse-PSSM features outperform others.

### Evaluation of the ensemble techniques

The performance of the first ensemble approach, *all voting*, on the training dataset is presented in Table 3. Since the data are balanced, we focused on the accuracy when comparing the performance of the different models. Among the seven ensembles in *all voting*, the SVM-based ensemble achieved the highest accuracy of 90.15%. The OET-KNN V500 ensemble, which reflects the performance of MemType-2L [2] on *DS-M*, achieved the second highest accuracy of 89.86%.

To choose the optimal feature set for *selective voting*, we tested the mRMR top-ranked *c* ($1\le c\le 50$) features incrementally by adding one feature at a time to the OET-KNN V50, KNN V50, SVM, GBM, and RF models, and the top-ranked c ($1\le c\le 500$) features on the OET-KNN V500 and KNN V500 models. The optimal feature set is the one with the highest accuracy. As observed from Fig. 3, the accuracy peaked when the number of top-ranked components were 3, 5, 15, 11, 1 for the OET-KNN V50-, KNN V50-, SVM-, GBM-, and RF-based ensembles, respectively. In addition, the optimal number of features for the OET-KNN V500 and KNN V500 ensembles were 20 and 21, respectively, as shown in Fig. 4; the performance started to deteriorate as more votes were counted. The detailed performances of the optimal feature set are presented in Table 4.

The results show that the ensemble models outperform their constituent classifiers, and the *selective voting* ensemble approach outperforms the *all voting* approach. Generally, the ensemble works the best when the individual classifiers making up the ensemble are both accurate and have low correlation [42, 43]. The superiority of *selective voting* over *all voting* is due to mRMR method ability to choose the models that have low correlation among each other and high correlation with the target class (i.e., most accurate), and the incremental feature selection ability to select the optimal set that reduces the noise and increases the ensemble classifier distinctive power. An interesting observation to note here is that RF classifiers did not show improvement with ensemble approaches—since the optimal number of features was only one. This indicates that since the RF model is an ensemble, it is more robust and consistent than the other models, although not necessarily the most accurate. Further, while the individual SVM and GBM classifiers generally provided higher performances than those of the OET-KNN and KNN classifiers, the latter leveraged more from the *selective voting* ensemble. This suggests that the predictions from the OET-KNN and KNN classifiers are less consistent (i.e., they make errors in different parts of the input space) and are therefore better candidates for the ensemble than the SVM and GBM classifiers.

The best performance in all methods was achieved by *selective voting* with the OET-KNN V500 ensemble, where the overall accuracy reached 91.31%, which is 1.67% higher than what the MemType-2L method (OET-KNN V500 with *all voting*) achieved. Because it achieved the best performance, the *selective voting* approach with the OET-KNN V500 method is utilized in the integrative approach *TooT-M*.

### Evaluation of transmembrane topology prediction tools

The performances of HMMTOP [31], TMHMM [32], TOPCONS2 [8] and PRED-TMBB2 [22] on the *DS-M* dataset are shown in Table 5. Based on statistics in the dataset section, we expected the topology prediction tools to fail to predict at least 20% of the membrane proteins because they are not transmembrane proteins; the results reported here confirm this hypothesis. The transmembrane topology reached a maximum sensitivity of 72%. This finding further highlights the importance of building a model to predict all membrane types and that transmembrane topology tools disregard surface-bound proteins and thus fail to recognize more than 20% of membrane proteins. Nevertheless, a very attractive aspect here is the exceptionally high specificity (true negative rate) in TOPCONS2 predictions, which is due its ability to distinguish signal peptides from transmembrane regions [9]. This property means that the confidence in the positive prediction is high; thus, this aspect is exploited in *TooT-M*.

### Performance of integrative approach *TooT-M*

The integrative approach *TooT-M* combines the best models from both the transmembrane topology tools (TOPCONS2) and the all-type membrane predictors (*selective voting* OET-KNN V500) through weighted voting. In weighted voting, a positive vote from TOPCONS2 is trusted and multiplied by the number of constituent classifiers in the *selective voting* OET-KNN V500 ensemble minus one; that is, the OET-KNN V500 *selective voting* prediction is transformed to positive *if and only if* there is at least one constituent classifier that agrees with the positive prediction of TOPCONS2. Among all the tested weights, this approach helped enhance the sensitivity without negatively impacting the specificity.

Table 6 shows the LOOCV performance of *TooT-M*. Compared to the *selective voting* OET-KNN V500 ensemble, the sensitivity (true-positive rate) was enhanced by 2.76% and the specificity was enhanced by 1.35%. Overall, the accuracy increased by 2%, and the MCC was boosted by 4%.

### Comparison with the state-of-the-art methods

Here we compare the performance of *TooT-M* to the state-of-the-art methods in three settings:

1. The *TooT-M*, Mem-2LSAAC [3], and MemType-2L [2] methods are trained on the *DS-M* training set, and their performances are evaluated on the *DS-M* testing set.
2. The *TooT-M* method is trained on the dataset obtained by the iMem-2LSAAC authors (DS1), and its performance is compared with the reported performance of iMem-2LSAAC [3] on the same dataset.
3. The *TooT-M* method is trained on the dataset provided by Chou and Shen [2] (DS2), and its performance is compared to the reported performance of MemType-2L [2] on the same dataset.

As illustrated in Fig. 5 and indicated in Table 7, the integrative approach outperformed all of the other methods in terms of sensitivity, specificity, accuracy, and MCC. In addition, the integrative approach achieved receiver operating characteristic area under the curve of 0.97 compared to 0.95 and 0.82 by the state-of-the art, as shown in Fig. 6.

Similarly, as shown in Table 8, it outperformed Mem-2LSAAC [3] in terms of specificity, accuracy, and MCC, while still keeping the sensitivity credible. It also outperformed MemType-2L [2] in terms of sensitivity, accuracy, and MCC, while achieving a similar specificity, as shown in Table 9.

## Conclusion

We curated a new membrane protein benchmark dataset that contains all types of membrane proteins, including surface-bound proteins. We demonstrated the limitation of using only transmembrane topology prediction tools to predict all types of membrane proteins, as they detect only transmembrane proteins and miss surface-bound proteins, which account for approximately 20% of membrane protein data. Furthermore, we evaluated the performances of different protein-encoding techniques, including those employed by the state-of-the-art membrane predictors with different machine learning algorithms. The experimental results obtained by cross-validation and independent testing suggest that applying an integrative approach that combines the results of transmembrane topology prediction and Pse-PSSM OET-KNN predictors yields the best performance. *TooT-M* achieved a 92.51% accuracy in independent testing, compared to the 89.53% and 79.42% accuracies achieved by the state-of-the-art methods MemType-2L [2] and iMem-2LSAAC [3], respectively.

## Supplementary information

**Additional file 1.** Detailed performance evaluation of Pse-PSSM where *λ* ∈ (0, …, 49)

**Additional file 2.** Detailed performance of five-fold, and ten-fold cross-validation

## Data Availability

TooT-M is available at: https://github.com/bioinformatics-group/TooT-M

## Abbreviations

AAC: Amino acid composition
AUC: Area-under-curve
GBM: Gradient-boosting machine
GPI: Glycophosphatidylinositol
IMP: Integral membrane protein
KNN: k-nearest neighbor
LOOCV: Leave-one-out cross-validation
MI: Mutual information
MID: Mutual information difference
mRMR: Minimum redundancy maximum relevance
OET-KNN: Optimized evidence-theoretic k nearest neighbor
PAAC: Pair amino acid composition
Pse-PSSM: Position-specific scoring matrix
PseAAC: Pseudo-amino acid composition
RF: Random forest
ROC: Receiver operating characteristic
SAAC: Split amino acid composition
SVM: Support vector machine
TMS: Transmembrane segment
