# Supplementary material for: Integrative approach for detecting membrane proteins
Source: BMC Bioinformatics. 2020 Dec 21;21(Suppl 19):575. doi: 10.1186/s12859-020-03891-x (PMC7751106; doi:10.1186/s12859-020-03891-x)
Supplement: Supplementary file 1 — Additional file 1. Detailed performance evaluation of Pse-PSSM where λ ∈ (0, …, 49) [file 12859_2020_3891_MOESM1_ESM.pdf]

5-Fold cross validation and 10-Fold cross validation performances

Table 1 shows the 5-Fold cross-validation performance of individual models.

Table 1: 5-Fold cross-validation performance of the individual models

Encoding	ML Algorithm	Sensitivity	Specificity	Accuracy	MCC
AAC	OET-KNN	71.53	79.31	75.47	0.51
	KNN	67.01	84.01	75.62	0.52
	SVM	71.66	82.82	77.31	0.55
	GBM	70.72	83.07	76.98	0.54
	RF	68.12	84.97	76.66	0.54
PAAC	OET-KNN	69.57	80.10	74.90	0.50
	KNN	65.59	81.07	73.43	0.47
	SVM	76.79	84.15	80.52	0.61
	GBM	73.92	84.75	79.41	0.59
	RF	71.62	81.65	76.70	0.54
PseAAC	OET-KNN	74.14	78.85	76.52	0.53
	KNN	69.30	83.49	76.48	0.53
	SVM	70.90	83.79	77.42	0.55
	GBM	72.91	82.97	78.00	0.56
	RF	68.42	84.81	76.71	0.54
SAAC	OET-KNN	70.60	68.87	69.72	0.39
	KNN	63.91	76.01	70.04	0.40
	SVM	74.97	83.61	79.34	0.59
	GBM	73.35	84.76	79.13	0.59
	RF	68.04	87.24	77.77	0.56
Pse-PSSM, $\lambda = 0$	OET-KNN	86.27	88.44	87.37	0.75
	KNN	79.58	93.18	86.47	0.74
	SVM	82.81	90.12	86.51	0.73
	GBM	81.80	90.18	86.05	0.72
	RF	78.99	92.33	85.75	0.72
Pse-PSSM, $\lambda = 1$	OET-KNN	86.05	88.30	87.19	0.74
	KNN	80.52	91.97	86.32	0.73
	SVM	86.19	91.88	89.08	0.78
	GBM	83.69	91.53	87.66	0.76
	RF	79.22	93.22	86.31	0.73
Pse-PSSM, $\lambda = 2$	OET-KNN	85.83	87.85	86.85	0.74
	KNN	80.38	91.04	85.78	0.72
	SVM	86.13	91.77	88.99	0.78
	GBM	83.98	91.50	87.79	0.76
	RF	79.03	93.45	86.34	0.73

This table shows microaverage 5 Fold cross-validation performance of the different protein encodings on different machine learning algorithms.

Table 2 shows the 10-Fold cross-validation performance of individual models.

Table 2: 10-Fold cross-validation performance of the individual models

Encoding	ML Algorithm	Sensitivity	Specificity	Accuracy	MCC
AAC	OET-KNN	71.54	78.58	75.11	0.50
	KNN	67.26	83.96	75.72	0.52
	SVM	72.05	82.78	77.48	0.55
	GBM	70.5	83.5	77.08	0.54
	RF	68.48	84.84	76.77	0.54
PAAC	OET-KNN	73.08	77.93	75.54	0.51
	KNN	68.65	82.27	75.55	0.51
	SVM	70.89	84.06	77.56	0.55
	GBM	72.11	82.48	77.36	0.54
	RF	68.53	84.44	76.59	0.53
PseAAC	OET-KNN	69.91	80.07	75.05	0.50
	KNN	66.08	80.91	73.59	0.47
	SVM	76.6	84.36	80.53	0.61
	GBM	74.02	84.88	79.52	0.59
	RF	71.23	81.39	76.38	0.52
SAAC	OET-KNN	71.06	68.9	69.97	0.39
	KNN	64.22	76.41	70.39	0.41
	SVM	74.86	84.26	79.62	0.59
	GBM	73.52	84.99	79.33	0.59
	RF	67.97	87.05	77.64	0.56
Pse-PSSM, $\lambda = 0$	OET-KNN	86.23	88.35	87.31	0.74
	KNN	79.74	93.49	86.7	0.74
	SVM	83.08	90.4	86.79	0.73
	GBM	81.88	90.38	86.18	0.72
	RF	79.17	92.35	85.85	0.72
Pse-PSSM, $\lambda = 1$	OET-KNN	86.31	89.05	87.7	0.75
	KNN	80.94	92.31	86.7	0.73
	SVM	87	92.06	89.56	0.79
	GBM	83.98	91.46	87.77	0.75
	RF	79.6	93.55	86.67	0.74
Pse-PSSM, $\lambda = 2$	OET-KNN	86.02	88.39	87.22	0.74
	KNN	81.03	91.87	86.52	0.73
	SVM	86.78	92.09	89.47	0.79
	GBM	84.33	92.2	88.32	0.76
	RF	79.71	93.54	86.72	0.74

This table shows microaverage 10 Fold cross-validation performance of the different protein encodings on different machine learning algorithms.
