# Supplementary material for: Integrative approach for detecting membrane proteins
Source: BMC Bioinformatics. 2020 Dec 21;21(Suppl 19):575. doi: 10.1186/s12859-020-03891-x (PMC7751106; doi:10.1186/s12859-020-03891-x)
Supplement: Supplementary file 2 — Additional file 2. Detailed performance of five-fold, and ten-fold cross-validation [file 12859_2020_3891_MOESM2_ESM.pdf]

Detailed performance evaluation of Pse-PSSM

Fig 1 delineates the accuracy of different models trained using the Pse-PSSM encodings ($\lambda \in (0, \ldots, 49)$).

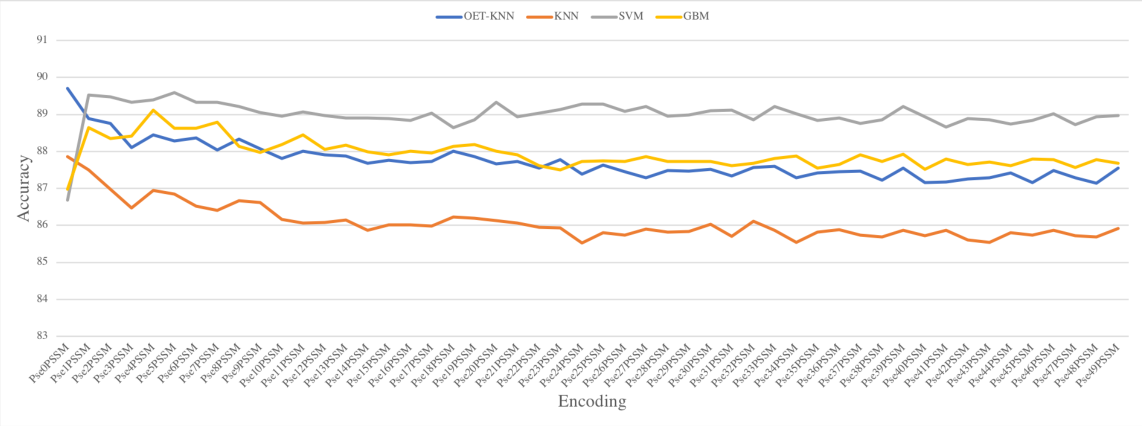

Figure 1: Choice of different models with Pse-PSSM, $\lambda \in (0, \ldots, 49)$

Table 1 shows the the detailed performance of Pse-PSSM where ($\lambda \in (0, \ldots, 49)$) in combination with different machine learning algorithms.

Table 1: LOOCV performances of the individual Pse-PSSM models

Encoding	ML Algorithm	Sensitivity	Specificity	Accuracy	MCC
Pse-PSSM, λ=0	OET-KNN	86.57	92.75	89.7	0.7953
	KNN	85.22	90.44	87.86	0.7580
	SVM	83.23	90.05	86.68	0.7350
	GBM	83.41	90.45	86.98	0.7409
Pse-PSSM, λ=1	OET-KNN	85.92	91.79	88.89	0.7788
	KNN	85.89	89.06	87.50	0.7501
	SVM	86.75	92.22	89.52	0.7912
	GBM	85.00	92.19	88.64	0.7744
Pse-PSSM, λ=2	OET-KNN	85.51	91.9	88.75	0.7762
	KNN	85.65	88.28	86.98	0.7397
	SVM	86.83	92.06	89.48	0.7904
	GBM	84.86	91.72	88.34	0.7682
Pse-PSSM, λ= 3	OET-KNN	84.69	91.44	88.11	0.7636
	KNN	84.97	87.92	86.47	0.7295
	SVM	86.97	91.61	89.32	0.7871
	GBM	85.01	91.74	88.42	0.7697
Pse-PSSM, λ=4	OET-KNN	85.46	91.37	88.45	0.7701
	KNN	85.44	88.41	86.95	0.7390
	SVM	86.87	91.85	89.39	0.7886
	GBM	85.71	92.41	89.11	0.7835
Pse-PSSM, λ=5	OET-KNN	85.17	91.32	88.29	0.7668
	KNN	85.60	88.07	86.85	0.7371
	SVM	86.82	92.26	89.58	0.7925
	GBM	85.10	92.08	88.63	0.7742
Pse-PSSM, λ=6	OET-KNN	85.26	91.39	88.37	0.7684
	KNN	85.41	87.61	86.52	0.7305
	SVM	86.72	91.85	89.32	0.7871
	GBM	84.96	92.22	88.63	0.7743
Pse-PSSM, λ=7	OET-KNN	84.95	91.06	88.04	0.762
	KNN	85.25	87.53	86.41	0.7281
	SVM	86.80	91.79	89.32	0.7872
	GBM	85.36	92.12	88.78	0.7771
Pse-PSSM, λ=8	OET-KNN	85.3	91.28	88.33	0.7676
	KNN	85.45	87.86	86.67	0.7335
	SVM	86.33	92.03	89.22	0.7853
	GBM	84.57	91.60	88.13	0.7641
Pse-PSSM, λ=9	OET-KNN	84.81	91.25	88.07	0.7626
	KNN	85.40	87.79	86.61	0.7322
	SVM	86.28	91.75	89.05	0.7819
	GBM	83.89	91.95	87.97	0.7614
Pse-PSSM, λ=10	OET-KNN	84.73	90.81	87.81	0.7572
	KNN	85.22	87.08	86.16	0.7232
	SVM	86.36	91.47	88.95	0.7796
	GBM	84.46	91.84	88.19	0.7655
Pse-PSSM, λ=11	OET-KNN	84.92	91.00	88.00	0.7611
	KNN	85.01	87.10	86.07	0.7214
	SVM	86.31	91.75	89.06	0.7821
	GBM	84.69	92.10	88.45	0.7707

LOOCV performances of the individual Pse-PSSM models (cont.)

Encoding	ML Algorithm	Sensitivity	Specificity	Accuracy	MCC
Pse-PSSM, λ=12	OET-KNN	84.59	91.11	87.90	0.7591
	KNN	85.00	87.13	86.08	0.7215
	SVM	86.26	91.59	88.96	0.7800
	GBM	84.24	91.77	88.06	0.7629
Pse-PSSM, λ=13	OET-KNN	84.80	90.87	87.87	0.7585
	KNN	84.67	87.57	86.14	0.7229
	SVM	86.42	91.32	88.90	0.7787
	GBM	84.62	91.60	88.16	0.7646
Pse-PSSM, λ=14	OET-KNN	84.54	90.72	87.67	0.7545
	KNN	84.92	86.78	85.87	0.7173
	SVM	86.49	91.27	88.91	0.7789
	GBM	84.33	91.55	87.99	0.7613
Pse-PSSM, λ=15	OET-KNN	84.64	90.79	87.76	0.7562
	KNN	84.56	87.45	86.02	0.7205
	SVM	86.22	91.49	88.89	0.7786
	GBM	84.32	91.42	87.91	0.7598
Pse-PSSM, λ=16	OET-KNN	84.66	90.66	87.70	0.7549
	KNN	85.15	86.87	86.02	0.7204
	SVM	86.03	91.55	88.83	0.7774
	GBM	84.15	91.75	88.00	0.7618
Pse-PSSM, λ=17	OET-KNN	84.82	90.56	87.73	0.7555
	KNN	84.97	86.96	85.98	0.7195
	SVM	86.56	91.46	89.04	0.7814
	GBM	84.51	91.30	87.95	0.7603
Pse-PSSM, λ=18	OET-KNN	84.64	91.30	88.01	0.7616
	KNN	84.72	87.70	86.23	0.7247
	SVM	86.17	91.05	88.64	0.7735
	GBM	84.48	91.68	88.13	0.7641
Pse-PSSM, λ=19	OET-KNN	84.96	90.70	87.86	0.7582
	KNN	85.45	86.91	86.19	0.7237
	SVM	86.07	91.55	88.85	0.7778
	GBM	84.68	91.60	88.19	0.7652
Pse-PSSM, λ=20	OET-KNN	84.46	90.78	87.66	0.7543
	KNN	84.76	87.47	86.13	0.7227
	SVM	86.36	92.23	89.33	0.7876
	GBM	84.17	91.76	88.01	0.762
Pse-PSSM, λ=21	OET-KNN	84.58	90.79	87.73	0.7556
	KNN	84.59	87.48	86.06	0.7212
	SVM	85.85	91.92	88.93	0.7796
	GBM	83.88	91.81	87.90	0.7598
Pse-PSSM, λ=22	OET-KNN	84.12	90.89	87.55	0.7523
	KNN	84.41	87.43	85.94	0.7189
	SVM	86.15	91.85	89.04	0.7817
	GBM	83.50	91.63	87.62	0.7543
Pse-PSSM, λ=23	OET-KNN	84.71	90.76	87.77	0.7565
	KNN	84.72	87.10	85.93	0.7186
	SVM	86.12	92.07	89.13	0.7837
	GBM	83.64	91.27	87.50	0.7518

LOOCV performances of the individual Pse-PSSM models (cont.)

Encoding	ML Algorithm	Sensitivity	Specificity	Accuracy	MCC
Pse-PSSM, λ=24	OET-KNN	84.61	90.08	87.38	0.7484
	KNN	84.33	86.69	85.52	0.7105
	SVM	86.34	92.13	89.27	0.7865
	GBM	83.69	91.65	87.72	0.7564
Pse-PSSM, λ=25	OET-KNN	84.15	91.01	87.63	0.7539
	KNN	84.51	87.07	85.80	0.7161
	SVM	86.44	92.04	89.28	0.7865
	GBM	83.79	91.59	87.74	0.7567
Pse-PSSM, λ=26	OET-KNN	84.34	90.47	87.45	0.75
	KNN	84.52	86.92	85.73	0.7147
	SVM	86.33	91.76	89.08	0.7825
	GBM	83.83	91.50	87.72	0.7561
Pse-PSSM, λ=27	OET-KNN	84.32	90.19	87.29	0.7468
	KNN	84.63	87.14	85.90	0.7181
	SVM	86.18	92.15	89.21	0.7852
	GBM	83.79	91.80	87.85	0.7589
Pse-PSSM, λ=28	OET-KNN	84.39	90.49	87.48	0.7506
	KNN	84.47	87.13	85.82	0.7164
	SVM	85.83	91.99	88.95	0.7802
	GBM	83.74	91.59	87.72	0.7562
Pse-PSSM, λ=29	OET-KNN	84.14	90.70	87.46	0.7504
	KNN	84.58	87.04	85.83	0.7166
	SVM	85.97	91.91	88.98	0.7806
	GBM	83.85	91.52	87.73	0.7565
Pse-PSSM, λ=30	OET-KNN	84.19	90.74	87.51	0.7514
	KNN	84.54	87.48	86.03	0.7208
	SVM	86.24	91.86	89.09	0.7827
	GBM	83.70	91.65	87.73	0.7565
Pse-PSSM, λ=31	OET-KNN	84.25	90.34	87.34	0.7477
	KNN	84.56	86.83	85.71	0.7142
	SVM	86.05	92.08	89.11	0.7832
	GBM	83.69	91.44	87.62	0.7541
Pse-PSSM, λ=32	OET-KNN	84.23	90.83	87.57	0.7527
	KNN	84.93	87.25	86.11	0.7222
	SVM	85.99	91.64	88.85	0.778
	GBM	83.75	91.48	87.67	0.7551
Pse-PSSM, λ=33	OET-KNN	84.28	90.84	87.60	0.7533
	KNN	84.32	87.39	85.87	0.7175
	SVM	86.18	92.15	89.21	0.7852
	GBM	84.02	91.50	87.81	0.7579
Pse-PSSM, λ=34	OET-KNN	84.02	90.46	87.28	0.7468
	KNN	84.25	86.78	85.54	0.7108
	SVM	86.17	91.77	89.01	0.7811
	GBM	83.69	91.96	87.88	0.7597
Pse-PSSM, λ=35	OET-KNN	84.30	90.45	87.42	0.7494
	KNN	84.67	86.93	85.82	0.7163
	SVM	85.75	91.84	88.83	0.7778
	GBM	83.74	91.23	87.54	0.7524

LOOCV performances of the individual Pse-PSSM models (cont.)

**Encoding**	**ML Algorithm**	**Sensitivity**	**Specificity**	**Accuracy**	**MCC**
Pse-PSSM, λ=36	OET-KNN	84.15	90.67	87.45	0.7503
	KNN	84.96	86.78	85.88	0.7176
	SVM	85.92	91.80	88.90	0.7789
	GBM	83.70	91.50	87.65	0.7549
Pse-PSSM, λ=37	OET-KNN	84.28	90.57	87.47	0.7504
	KNN	84.56	86.89	85.74	0.7148
	SVM	85.58	91.84	88.75	0.7761
	GBM	84.03	91.68	87.90	0.7598
Pse-PSSM, λ=38	OET-KNN	84.18	90.18	87.22	0.7453
	KNN	84.53	86.81	85.69	0.7137
	SVM	85.93	91.72	88.86	0.7783
	GBM	83.83	91.53	87.73	0.7563
Pse-PSSM, λ=39	OET-KNN	84.62	90.40	87.55	0.7519
	KNN	85.00	86.71	85.87	0.7173
	SVM	86.14	92.22	89.22	0.7855
	GBM	83.98	91.76	87.92	0.7603
Pse-PSSM, λ=40	OET-KNN	84.08	90.17	87.16	0.7443
	KNN	84.46	86.94	85.72	0.7144
	SVM	86.03	91.76	88.93	0.7796
	GBM	83.55	91.38	87.52	0.7522
Pse-PSSM, λ=41	OET-KNN	83.85	90.40	87.17	0.7446
	KNN	84.66	87.03	85.86	0.7172
	SVM	85.46	91.77	88.66	0.7744
	GBM	83.85	91.64	87.80	0.7578
Pse-PSSM, λ=42	OET-KNN	84.19	90.24	87.26	0.7461
	KNN	84.14	87.04	85.61	0.7123
	SVM	85.94	91.75	88.88	0.7787
	GBM	83.37	91.81	87.65	0.7551
Pse-PSSM, λ=43	OET-KNN	84.04	90.45	87.29	0.7469
	KNN	84.37	86.69	85.54	0.7109
	SVM	85.73	91.92	88.86	0.7784
	GBM	83.83	91.49	87.71	0.756
Pse-PSSM, λ=44	OET-KNN	84.24	90.50	87.41	0.7493
	KNN	84.78	86.80	85.80	0.7160
	SVM	85.88	91.53	88.74	0.7757
	GBM	83.56	91.55	87.61	0.7541
Pse-PSSM, λ=45	OET-KNN	84.02	90.22	87.16	0.7442
	KNN	84.56	86.89	85.74	0.7148
	SVM	85.80	91.77	88.83	0.7776
	GBM	83.83	91.66	87.80	0.7578
Pse-PSSM, λ=46	OET-KNN	84.32	90.56	87.48	0.7507
	KNN	84.51	87.20	85.87	0.7175
	SVM	85.97	91.99	89.02	0.7815
	GBM	83.73	91.70	87.77	0.7572
Pse-PSSM, λ=47	OET-KNN	84.03	90.47	87.29	0.747
	KNN	84.48	86.93	85.72	0.7145
	SVM	85.71	91.65	88.72	0.7755
	GBM	83.54	91.49	87.57	0.7532
Pse-PSSM, λ=48	OET-KNN	83.85	90.34	87.14	0.7439
	KNN	84.47	86.88	85.69	0.7138
	SVM	85.81	91.97	88.93	0.7798
	GBM	83.73	91.70	87.77	0.7572
Pse-PSSM, λ=49	OET-KNN	84.19	90.82	87.55	0.7522
	KNN	84.43	87.35	85.91	0.7183
	SVM	85.93	91.92	88.96	0.7803
	GBM	83.69	91.58	87.68	0.7556
